# Implementing medication reconciliation from the planner’s perspective: a qualitative study

**DOI:** 10.1186/1472-6963-14-290

**Published:** 2014-07-04

**Authors:** Sadie H Sanchez, Sanjum S Sethi, Susan L Santos, Kenneth Boockvar

**Affiliations:** 1 Icahn School of Medicine at Mount Sinai, One Gustave L. Levy Place, Box 1057, New York, NY 10029, USA; 2 Boston University School of Medicine, Division of Cardiology, 88 E Newton St, C-818, Boston, MA 02118, USA; 3 VA New Jersey Health Care System, War Related Illness and Injury Study Center East Orange, 385 Tremont Ave, Mailstop 129, East Orange, NJ 07018, USA; 4 Rutgers University School of Public Health Piscataway, Piscataway, NJ, USA; 5 Geriatrics Research, Education, and Clinical Center, James J. Peters Veterans Affairs Medical Center, 130 West Kingsbridge Road, Bronx, NY 10468, USA; 6 Jewish Home Lifecare Research Institute on Aging, New York, NY, USA

**Keywords:** Medication reconciliation, Adverse drug event, Patient safety, Implementation, National patient safety goals

## Abstract

**Background:**

Medication reconciliation can reduce adverse events associated with prescribing errors at transitions between sites of care. Though a U.S. Joint Commission National Patient Safety Goal since 2006, at present organizations continue to have difficulty implementing it. The objective of this study was to examine medication reconciliation implementation from the perspective of individuals involved in the planning process in order to identify recurrent themes, including facilitators and barriers, that might inform other organizations’ planning and implementation efforts.

**Methods:**

We performed semi-structured interviews with individuals who had a role in planning medication reconciliation implementation at a large urban academic medical center in the U.S. and its affiliated Veterans Affairs hospital. We queried respondents’ perceptions of the implementation process and their experience with facilitators and barriers. Transcripts were coded and analyzed using a grounded theory approach. The themes that emerged were subsequently categorized using the Consolidated Framework for Implementation Research (CFIR).

**Results:**

There were 13 respondents, each with one or more organizational roles in quality improvement, information technology, medication safety, and education. Respondents described a resource- and time- intensive medication reconciliation planning process. The planning teams’ membership and functioning were recognized as important factors to a successful planning process. Implementation was facilitated by planners’ understanding of the principles of performance improvement, in particular, fitting the new process into the workflow of multiple disciplines. Nevertheless, a need for significant professional role changes was recognized. Staff training was recognized to be an important part of roll-out, but training had several limitations. Planners monitored compliance to help sustain the process, but acknowledged that this did not ensure that medication reconciliation actually achieved its primary goal of reducing errors. Study findings fit multiple constructs in the CFIR model.

**Conclusions:**

Study findings suggest that to improve the likelihood of a successful implementation of medication reconciliation, planners should, among other considerations, involve a multidisciplinary planning team, recognize the significant professional role changes that may be needed, and consider devoting resources not just to compliance monitoring but also to monitoring of the process’ impact on prescribing.

## Background

Over the last decade, patient safety in the healthcare system has received increased attention from the medical community. An Institute of Medicine report from 2000 highlighted that 44,000-98,000 deaths per year could be attributed to preventable errors in healthcare delivery [1]. Observational studies have found that adverse events occur in 2-4% of hospitalized patients [2]. Of these, drug complications are the most common type, with 20% or more being medication-related; i.e., adverse drug events (ADEs) [3,4].

Evidence suggests that medication reconciliation can reduce ADEs associated with transitions within and between healthcare settings, especially for patients who are at higher risk because of older age, multiple chronic conditions, or use of many medications [5-7]. Medication reconciliation is the process of creating a best possible list of medications being used by a patient and comparing that list with the provider’s admission, transfer, and/or discharge orders. This occurs in 3 steps: verification (collecting the patient’s medication history), clarification (ensuring that the medications and doses are appropriate), and reconciliation (documenting changes in the orders) [8,9]. Since nearly 30% of prescribing errors are associated with incomplete medication histories on admission [10], a focus of this process is to reduce prescribing errors at hospital admission and discharge. In one study, medication reconciliation was shown to decrease ADEs caused by admission prescribing errors by 43% [11]. A systematic review suggests that the most effective medication reconciliation interventions are those that utilize pharmacy staff and focus on patients at high risk for adverse events [6].

The Joint Commission, an independent non-profit accreditation body of hospital systems, introduced medication reconciliation as a National Patient Safety Goal in 2006 [8]. Despite an extensive effort on the part of many organizations to implement it within various healthcare settings, implementation has been challenging [12]. After temporarily removing medication reconciliation from the list of National Patient Safety Goals, The Joint Commission brought it back again in 2011. Within this background, the objective of this study was to perform a qualitative examination of the medication reconciliation planning process in two healthcare organizations. We report on factors affecting implementation from the perspectives of those involved in planning, including physician managers, nurse managers, quality specialists, pharmacy managers, information technologists. We also indicate how emergent themes fit into constructs in a healthcare implementation model [13] and discuss how these findings could to inform organizations’ ongoing implementation efforts.

## Methods

### Study design

This qualitative study utilized key informant interviews with individuals involved in planning medication reconciliation implementation at two healthcare organizations, and used a grounded theory approach [14]. We chose to use individual semi-structured interviews as opposed to closed-ended survey questions to better explore respondents’ perspectives. Individual interviews were utilized in favor of focus groups to foster respondents’ freedom of expression without self-censoring.

### Setting and timing

The settings were a large urban academic tertiary care center and an affiliated Veterans Affairs (VA) hospital in New York City. At the time of the study, the medication reconciliation planning and implementation process had taken place approximately three years prior (i.e., the inpatient medication reconciliation procedure had been in place for three years).

### Respondents

Respondents were recruited who were involved in the medication reconciliation planning process, selected from: pharmacy directors, chiefs of staff, nurse managers, quality improvement managers, information technology representatives, and physicians with administrative roles. Potential respondents were members of standing or ad hoc planning committees such as quality improvement, patient safety, information technology, and risk management committees, and were recruited by email and telephone. We used a snowball sampling strategy in which initial respondents were asked to refer us to additional potential respondents, and we continued recruiting respondents until there were no further individuals involved in the medication reconciliation planning process to interview. Respondents provided written informed consent, and the protocol was approved by the Institutional Review Boards of the James J. Peters VA Medical Center and Icahn School of Medicine at Mount Sinai in New York City.

### Data collection

Interviews took place in or near the respondents’ own offices and were conducted by two research team members. The primary interviewer (SLS) used an interview guide of semi-structured questions and probes while a second interviewer (KSB) asked additional clarifying questions. The interviewers explored respondents’ perceptions of the medication reconciliation implementation planning process and their attitudes regarding its purpose. They were questioned on the optimal roles in the process for physicians, nurses, pharmacists and other members of the healthcare team. They were asked about facilitators and barriers to planning and implementation and potential improvements to the process. Interviewers debriefed after each interview to identify additional potential areas of exploration and focus for subsequent interviews. Interviews were 30 to 60 minutes in length, were audiotaped, and were transcribed verbatim. Only one author (KSB) had access to the file linking transcripts with respondents’ identities.

### Data analysis

Transcripts were analyzed using a grounded theory approach [15-17]. Using multiple close readings, two research team members (SLS and KSB) reviewed the interviews independently to generate a list of concepts and domains and to determine a preliminary coding scheme. Codes were derived from a combination inductive approach using a constant comparison method [14]. To test the preliminary scheme, an initial transcript was independently coded by two investigators and the scheme revised. Discussion among all four investigators yielded a final coding scheme. Two team members (SHS and SSS) then applied the codes to each transcript by labeling individual words or phrases on hard copy. Consistency and reliability was repeatedly assessed by having the coders review a randomly selected set of transcript passages in duplicate and reconcile disagreement by discussion, with input from a third team member. The coded transcripts were entered into software Atlas.ti version 5.2 (Berlin, Germany) to facilitate sorting of passages. Research team members compared codes within and across interviews to elucidate themes. Quotations were chosen that were illustrative of each theme. Themes were then labeled using constructs in the Consolidated Framework for Implementation Research (CFIR), which was selected because it combines constructs across multiple published implementation theories [13]. This report adheres to the BioMed Central guideline (RATS) for reporting qualitative studies [18].

## Results

### Characteristics of respondents

Of 14 individuals invited to participate, one was unavailable to be interviewed. Of the 13 interview respondents 12 reported participating directly in the medication reconciliation planning process while one became involved in medication reconciliation after implementation was already underway. Respondents had the following department or committee roles: quality improvement (4), information technology (4), medication safety (3), and education (2). They had on average 5.9 (SD = 3.7) years of experience in their current position and all except one were present in their current position at the time the medication reconciliation implementation process had taken place. By professional training, there were four physicians, four nurses, four pharmacists, and one information technologist.

#### ***Theme 1***

Respondents recognized the value of medication reconciliation in improving patient safety and reducing medication errors, but respondents also had a broader view of its value that motivated them to contribute to the planning process.

Respondents indicated a high level of agreement that the purpose of medication reconciliation is to improve patient safety by preventing errors in medication history-taking, recording, and decision-making. Several respondents indicated that medication reconciliation has additional value in that it increases patient dialogue and education, promotes communication between healthcare providers, and mitigates risks at transfer points:

The purpose of medication reconciliation is in a global way to provide safe care because when the patient comes into the hospital we’ve come to realize we need to have a firm understanding of the medications the patients have been taking at home, and the importance of it has risen (Nurse Manager).

Respondents were motivated to participate in the medication reconciliation planning process by a belief that medication reconciliation is inherently proper medical management, not just because it is a Joint Commission standard: “This is one of the few things that is mandated that has a value” (Pharmacist Manager).

A few respondents observed that medication reconciliation also forces physicians to make individual active prescribing decisions for each and every medication and bring increased cognitive focus to their prescribing decisions:

It forces the provider to say is this drug therapy still consistent with the patient's current medical condition, should it be continued at this time or not, and should it be reinstituted when the patient goes home (Pharmacist Manager).

These quotes reflect planners’ knowledge and beliefs about medication reconciliation, and reflect the influence of such beliefs on the implementation planning process, a core CFIR construct (Table 1).

**Table 1:** **Themes, facilitators, and barriers to implementing medication reconciliation according to qualitative analysis of interviews with healthcare managers, and selected relevant Consolidated Framework for Implementation Research (CFIR) constructs **[13]

**Facilitator**	**Barrier**	**CFIR construct(s) with short definition **[13]
*Theme 1: Consensus that purpose of medication reconciliation is to improve patient safety; respondents also had a broader view of its value*		
Planners with a broad view of the process’ value		Individuals’ knowledge and beliefs about the intervention: “attitudes toward and value placed on the intervention as well as familiarity with facts, truths, and principles related to the intervention” [13]
		External policy and incentives: “external strategies to spread interventions including policy and regulations (governmental or other central entity), external mandates, … and public or benchmark reporting” [13]
*Theme 2: Planning team’s membership and functioning recognized as facilitators to a successful planning process*		
Planners who were or became champions of the process		Engaging champions: “’individuals who dedicate themselves to supporting, marketing, and “driving through” an [implementation]’, overcoming indifference or resistance that the intervention may provoke in an organization” [13]
Planners organizationally positioned to carry out the plan		Engaging individuals: “attracting and involving appropriate individuals in the implementation” [13]
Planners who were compelling leaders, who could get buy-in from front line staff		Engaging opinion leaders: “individuals in an organization who have formal or informal influence on the attitudes and beliefs of their colleagues with respect to implementing the intervention” [13]
Planners with openness to others’ perspectives and a willingness to compromise, to facilitate discussion and joint problem-solving		Learning climate: “climate in which leaders express their own fallibility and need for team members’ assistance and input; team members feel that they are essential, valued, and knowledgeable partners in the change process; individuals feel psychologically safe to try new methods; …sufficient time and space for reflective thinking and evaluation” [13]
Perseverance in obtaining resources	Lack of resources, staffing and/or budgetary support	Available resources: “the level of resources dedicated for implementation …including money, training, education, physical space, and time” [13]
Multi-departmental participation in planning		Process planning: “the degree to which scheme[s] … for implementing an intervention are developed in advance and the quality of those schemes” [13]
Communication among team members, in or out of meetings	Poor team communication	Networks and communications: “the nature and quality of formal and informal communications within an organization” [13]
*Theme 3: Implementation facilitated by planners’ understanding of performance improvement, and fitting the new process into workflow*		
Planners with an understanding of the basic tenets of performance improvement		Individuals’ other personal attributes: “personal traits such as tolerance of ambiguity, intellectual ability, motivation, values, competence, capacity, and learning style” [13]
Fitting the process into each discipline’s workflow		Compatibility: “how the intervention fits with existing workflows and systems” [13]
Assigning roles to multiple disciplines	Resistance to changing roles and/or scope of practice; enforcer is a negative role	Implementation climate: “The absorptive capacity for change, shared receptivity of involved individuals to an intervention” [13]
Providing value to front-line providers to improve uptake		Relative advantage: “stakeholders’ perception of the advantage of implementing the intervention versus an alternative solution” [13]
Testing to optimize human-computer usability	IT staff may not be available or able to do testing	Trialability: “ability to test the intervention on a small scale in the organization, and to be able to reverse course” [13]
Recognition that intervention should be refined based on reevaluation		Trialability (see above for definition)
*Theme 4: Training recognized as important to sustaining the process, but training has limited effect on some individuals or groups*		
Training all staff and tracking training	Staff turnover high; non-compliance not always solved by retraining	Available resources (see above for definition)
Marketing campaign with slogan		Networks and communications (see above for definition)
Successful training approaches: peer-to-peer training; didactic with real case examples		Individuals’ knowledge and beliefs about the intervention (see above for definition)
		Self-efficacy: “individuals’ belief in their own capabilities to execute course of action to achieve implementation goals” [13]
Trainees’ experiencing first hand avoided errors to drive home importance	Work and other activities compete for trainees’ attention	Individual stage of change: “characterization of the phase an individual is in, as he or she progresses toward skilled, enthusiastic, and sustained use of the intervention” [13]
		Relative priority: “Individuals’ perception of the importance of the implementation” [13]
*Theme 5: Planners monitored compliance to help sustain the process, but this did not ensure achievement of reduced errors*		
Monitoring of completion rates	Completion rates provide no data on health impact; lack of resources to gather such data	Executing: “carrying out or accomplishing the implementation according to plan” [13]
		Available resources (see above for definition)
Feedback of monitoring results to providers; fostering competition to increase compliance	Dilemma that error reports could go up if the new process results in more recognition	Reflecting and evaluating: “quantitative and qualitative feedback about the progress and quality of implementation accompanied with regular personal and team debriefing about progress and experience” [13]

#### ***Theme 2***

The planning teams’ membership and functioning were recognized as facilitators to a successful medication reconciliation planning process.

A majority of respondents agreed that the planning team had to be multidisciplinary; i.e., to include stakeholders in medical, nursing, pharmacy, and information technology departments, as well as relevant committee members (e.g. patient safety, quality improvement, and risk management) and administrators such as the Chief Medical Officer. Respondents indicated that implementation was facilitated by planning team members who were or became champions of the medication reconciliation process.

A key functional characteristic of the planning team was ease of communication. Respondents indicated openness to others’ perspectives and willingness to compromise, both of which facilitated discussion and joint problem-solving. Commitment of members to the planning process was high. Many respondents commented that, in the beginning, meetings were scheduled at least weekly in order to create momentum and facilitate rapid trouble-shooting. Open channels of communication outside of the meetings were equally important.

Implementation was facilitated by planning members who were perceived as compelling leaders and who could get cooperation from front line staff:

Buy-in is extremely important - getting principals and strong people on the committee. People who are good at convincing …. You have to have a provider onboard if you expect the residents and the other attendings to go with it (Pharmacist Manager).

Having organizationally well-positioned planning team members was important when it came to obtaining resources, which was often noted as a barrier. Statements indicated that planning team members persevered to obtain the necessary resources, even when faced with administrators who were reluctant to allocate them: “I know how to push some buttons and I probably pushed some buttons if I needed to [get administrators] onboard” (Pharmacist Manager). “There was no budget for this and there were no resources….. [So the project was accomplished by] whatever we could grab…. It was on our time” (Physician Manager).

These quotes underscore the importance of engaging champions, opinion leaders, and individuals; the organizational “learning climate”; available resources; and communications, all core CFIR constructs (Table 1), and their contribution to the medication reconciliation planning process.

#### ***Theme 3***

Planners’ approach to implementation was facilitated by understanding the need to fit the new process into workflow and to revise the process as needed, basic tenets of performance improvement.

Respondents recognized a need to fit the new medication reconciliation process into the hospital’s existing staffing structure and multiple disciplines’ different workflows (the CFIR construct of “compatibility”) (Table 1). Initially each discipline’s medication management workflow was examined, after which each discipline’s role in the new medication reconciliation process was proposed. Respondents noted that physicians were the ultimate decision makers and that completing medication reconciliation is part of their hospital admission process. However, each organization assigned varying roles to other disciplines. In one case, pharmacists were assigned to be present on the patient care units to educate the patients and talk with the doctors. This was considered to be a transformative role change:

We used to have pharmacists work centrally and verify the orders remotely and we changed the paradigm and sent pharmacists to the floors. So now… every unit has a pharmacist during the day (Pharmacist Manager).

Yet, the limits of the pharmacist’s new role was also articulated:

As a pharmacist, it's not in my scope of practice to decide this versus this. It's within my scope to make recommendation based on what I'm seeing but it's up to the provider in the end to make that final decision (Pharmacist Manager).

In another case of changing traditional roles, respondents decided that nurses could input medication information into the medication reconciliation record before the physicians did. This created a multi-disciplinary approach to the medication reconciliation process with the added unexpected, benefit of increased communication among all providers:

I think it promotes communication between the doctors and the nurses …. Many times patients tell nurses things they don’t tell doctors and vice versa (Nurse Manager).

Respondents expressed that increasing work efficiency was an important facilitator of uptake by providers:

We tried to offer something of value. For the physicians… no typing in meds or anything, its click, click, click, do a little editing and you get this beautiful discharge summary …. For the nurse, you don’t have to write down your own list on admission or on discharge, you just click, accept, and get what you need for patients…. I think that was pretty important to people that it helped them do the work they were already doing (Physician Manager).

Respondents also recognized that implementation is dynamic. They recognized that the process would be imperfect at the beginning and would need to be adapted. Several described an initial piloting in which they watched an early user navigate the process and then adjusted the process to better accommodate users (the CFIR construct of “trialability” (Table 1)).

#### ***Theme 4***

Staff training was recognized to be an important part of rolling out and sustaining the process, but training has limited effect on some individuals or groups.

Several training approaches were reported, including didactic presentations during staff orientation, online learning, presentations at departmental in-services and conferences, and one-to-one training by peers. Training occurred on all shifts and respondents reported the creation of tracking systems to ensure everyone involved was trained. Promotion of the new process among providers was facilitated by a marketing campaign with the slogan, “One list. One process. Universal access.”

Real near-miss case examples were presented to providers to demonstrate how medication reconciliation can affect outcomes. There was recognition of the importance of new users’ experiencing the benefit first hand in order to drive home the importance of complying with the new medication reconciliation process:

They don’t see a value until they come across the case where they picked up a mistake and then they say, ‘Oh thank God we had this because look at what I caught.’ [When] they've had that firsthand experience of capturing something, that brings it home to them (Pharmacist Manager).

Respondents also reported limitations to training approaches. First, staff turnover, especially of house staff and trainees, was high, and rotations short, resulting in insufficient time to solidify practice habits. Work and other training activities competed for staff attention. One respondent described an abbreviated “see one, do one, teach one” approach to the new medication reconciliation process. One respondent made the observation that lapses in compliance cannot always be attributed to inadequate training, nor corrected by more training:

Training is training. Performance is performance. There's a link but you've got to be very careful in always blaming training…. at some point the adult learner has to take training and use it…. You've got to hold people accountable (Nurse Manager).

These quotes reflect recognition that training’s impact depends on the educational phase an individual is in, an individual’s perception of the importance of medication reconciliation, and the concept of self-efficacy. This theme is consistent with the core CFIR construct of the influence of “individuals’ belief in their own capabilities to execute course of action to achieve implementation goals” (Table 1).

#### ***Theme 5***

Planners monitored compliance to help sustain the new process, but acknowledged that this did not ensure that medication reconciliation was actually achieving its primary goal of reducing errors.

Most respondents indicated that monitoring of completion rates helped ensure that providers were completing medication reconciliation appropriately. They agreed that the best way to ensure compliance was via direct feedback of monitoring data to providers, to hold providers accountable, and to enable managers to reach out to providers individually, part of the CFIR construct of “reflecting and evaluating” (Table 1). Another respondent believed that competition among units or teams could facilitate increased compliance.

However, many also respondents noted that completion rates do not provide information on the quality of the medication reconciliation process or error avoidance. The central problem, as described by one respondent, is that “We’re not collecting a gold standard medication list, and comparing it to what people enter into the system. We don’t have the infrastructure for that” (Physician Manager). This individual leaves open the question of whether execution (“carrying out or accomplishing the implementation according to plan,” a core CFIR construct, Table 1) has actually been achieved. Another respondent agreed:

We’re not … doing what really … should be happening which is … a continuous process where we’re gathering data in a consistent way and measuring our progress against it and deciding whether that data is meaningful for patient care. It’s easy to gather numbers – compliance – but whether or not that means patient’s lives are better, or errors are reduced, is much harder (Another Physician Manager).

Respondents acknowledged that ADE reports could go down if the medication reconciliation process was successful in preventing them, or, paradoxically, go up if the new process resulted in increased recognition and reporting of them, thereby creating a safety reporting dilemma.

## Discussion

In this study, we describe the implementation of medication reconciliation at two hospitals through analysis of semi-structured interviews with individuals instrumental in planning and implementing the process. Respondents agreed that the purpose of medication reconciliation is to improve patient safety by reducing medication errors and ensure compliance with the Joint Commission National Patient Safety Goal. Planning process characteristics emphasized by the respondents included: assembling an interdisciplinary team, getting buy-in from administrators and clinical leaders, fitting the new process into providers’ existing workflow, and providing value to providers assigned to key roles. Assignment of roles was a decision that required discussion and compromise during the planning process. Overall, key qualities of planning team members included a significant stake in the outcome, cooperative spirit and willingness to compromise and embracing challenges associated with solving a complex problem.

Results of the current study indicated that planners understood tenets of performance improvement, and followed steps akin to a Plan-Do-Study-Act (PDSA) model. PDSA is a change model consisting of the following steps: developing a plan to implement and test the change (Plan), carrying out the change (Do), observing and learning from the consequences (Study), and determining what modifications should be made (Act) [19]. Our study included statements that indicated acceptance of an imperfect process at the beginning followed by process refinement, fitting the process into existing workflow to the extent possible, use of teaching and marketing to generate acceptance of the process by users, and subsequent monitoring of and feedback on process performance. Use of a PDSA approach is notable because medication reconciliation may be more difficult to implement than other performance improvement interventions, as demonstrated by the history of pushback against the Joint Commission National Patient Safety Goal by many organizations [8]. Whether adaptation and promotion of the PDSA approach specifically for this process could improve its implementation is an open question.

Respondents indicated that there were some important role changes or adjustments that were expected of providers. For physicians, the introduction of medication reconciliation forced focused consideration of the patient’s entire list of medical problems and treatments. For pharmacists, transformative roles required pharmacists to spend more time on the hospital units reviewing medications with patients before discharge and interfacing with medical and nursing providers. This finding is consistent with other studies, which have reported on role changes of similar magnitudes for physicians, pharmacists, and nurses involving medication reconciliation such as having hospital nurses, pharmacists or pharmacy technicians play a larger role in taking medication histories [20,21]. Yet studies also report significant barriers to providers’ accomplishing the new tasks, including unreliable sources of medication information and tasks that compete for providers’ time and attention that they consider higher priority. As a result providers are at risk for having low self-efficacy; i.e., low perceived capability to achieve the process’ objectives [22].

Our study produced some unanticipated findings. First, although respondents were unanimous that the purpose medication reconciliation was to decrease errors, monitoring efforts focused on completion of the medication reconciliation process (i.e., compliance), with limited resources allocated to monitoring its accuracy or effectiveness (i.e., effect on medication errors). This is important because, in a previous study we found that providers who were completing medication reconciliation did not have confidence that the process was sufficient to prevent errors and ADEs as intended and their attitudes toward the process were often negative [22]. Other authors have noted the insufficiency of simply monitoring compliance [12] and at least one safety advocacy group has promoted a measure called the Medication Reconciliation Success Index (MRSI), which is a measure of avoidance of unintended discrepancies adjusted for the total number of pre-hospital medications [MRSI = 1-(number of unintended discrepancies/number of pre-hospital medications)] [23].

Second, the majority of the suggestions for improving the medication reconciliation process were hospital, provider, or departmental measures, and not driven by considering the patient’s experience. Patient-centered comments were rare, potentially in part because we did not ask about the patient experience. Yet improving the patient experience with medication reconciliation could have a positive impact on medication-related outcomes and patient satisfaction. One emerging patient-centered measurement tool, the Care Transitions Measure, explicitly asks patients or family members whether they received all the information needed to manage their medications upon discharge from the hospital [24]. Knowing the effect of medication reconciliation on health care utilization such as hospitalization is also important to patients, providers, and planners. Although studies to date have not shown a consistent effect, this may be because of the design of the medication reconciliation intervention studied or because other factors had greater influence on hospital utilization [6].

A strength of this study is that it follows our prior studies in same setting, and our interview script, coding, and analysis were informed by prior data and experience [22,25]. In this regard, a limitation of the study is that one author (KSB) was a member of the participating organizations during medication reconciliation planning and implementation, which may have influenced data collection and interpretation. However, this was not the case for the remaining authors. Results of this study were also shown to be consistent with constructs in an implementation model, the CFIR Table 1) [13]. Categories of constructs that fit our results include: intervention characteristics (e.g., relative advantage, trialability), inner setting (e.g., networks and communications, implementation climate, compatibility, relative priority, goals and feedback, learning climate, available resources), characteristics of individuals (e.g., knowledge and beliefs about the intervention, self-efficacy, individual stage of change), and process (e.g., planning, engaging opinion leaders, engaging champions, executing, and reflecting and evaluating). Consistency with this framework shows how medication reconciliation implementation is similar to other implementation problems, and also shows gaps in research on the topic that should be pursued in future studies. A limitation of our study is that we applied CFIR framework retrospectively, and there are areas that we did not purposefully query, such as structural characteristics of the organization.

## Conclusion

In this qualitative study, planners of medication reconciliation described resource-intensive planning and implementation, acknowledged multiple challenges involved in these processes, and revealed a good understanding of performance improvement principles. Our findings suggest approaches that may improve the likelihood of medication reconciliation implementation success, and include constructs from established implementation models (i.e., PDSA and CFIR). Planners also need to understand provider perceptions of the new process, especially providers’ perceived capability to achieve the process’ objectives. The following approaches may be particularly useful to improve the likelihood of successful planning and implementation of medication reconciliation:

1) Involve a multidisciplinary planning team consisting of highly-respected individuals who are open to change and compromise

2) Recognize that effective medication reconciliation may not be achieved by just refining existing practice and that significant professional role changes may be necessary, especially for nursing and pharmacy staff

3) Recognize the need to trial the new process and refine it

4) Recognize and promote other benefits of the process, such as enhanced communication between disciplines

5) Plan to devote resources not just to compliance monitoring but also to monitoring of the process’ impact on prescribing in order to be able to provide clinically meaningful evidence of the impact of the process to front-line providers

## Competing interests

The authors declare that they have no competing interests.

## Authors’ contributions

SHS and SSS conducted pilot interviews, developed and applied the coding scheme, assisted in analysis, and helped draft the manuscript. SLS drafted the interview script, led interviews, helped draft the coding scheme, assisted in analysis, and revised the manuscript for important intellectual content. KSB assisted in all interviews, drafted the interview script and coding scheme, and drafted the manuscript. All authors read and approved the final manuscript.

## Pre-publication history

The pre-publication history for this paper can be accessed here:

http://www.biomedcentral.com/1472-6963/14/290/prepub
